# Supplementary material for: Construction of a solid Cox model for AML patients based on multiomics bioinformatic analysis
Source: Front Oncol. 2022 Aug 10;12:925615. doi: 10.3389/fonc.2022.925615 (PMC9399435; doi:10.3389/fonc.2022.925615)
Supplement: Supplementary file 1 [file DataSheet_1.docx]

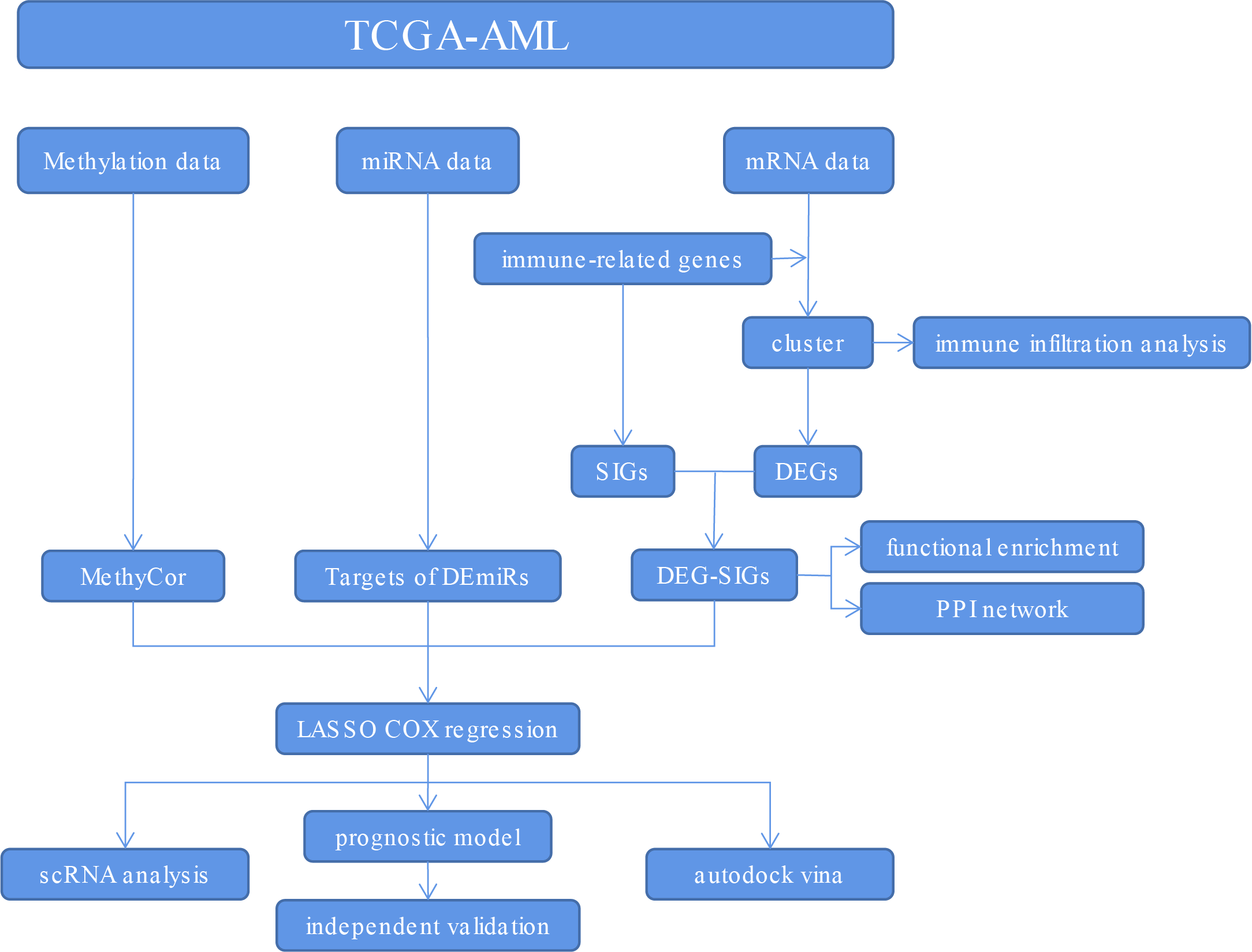


Figure S1. Flowchart of this study.


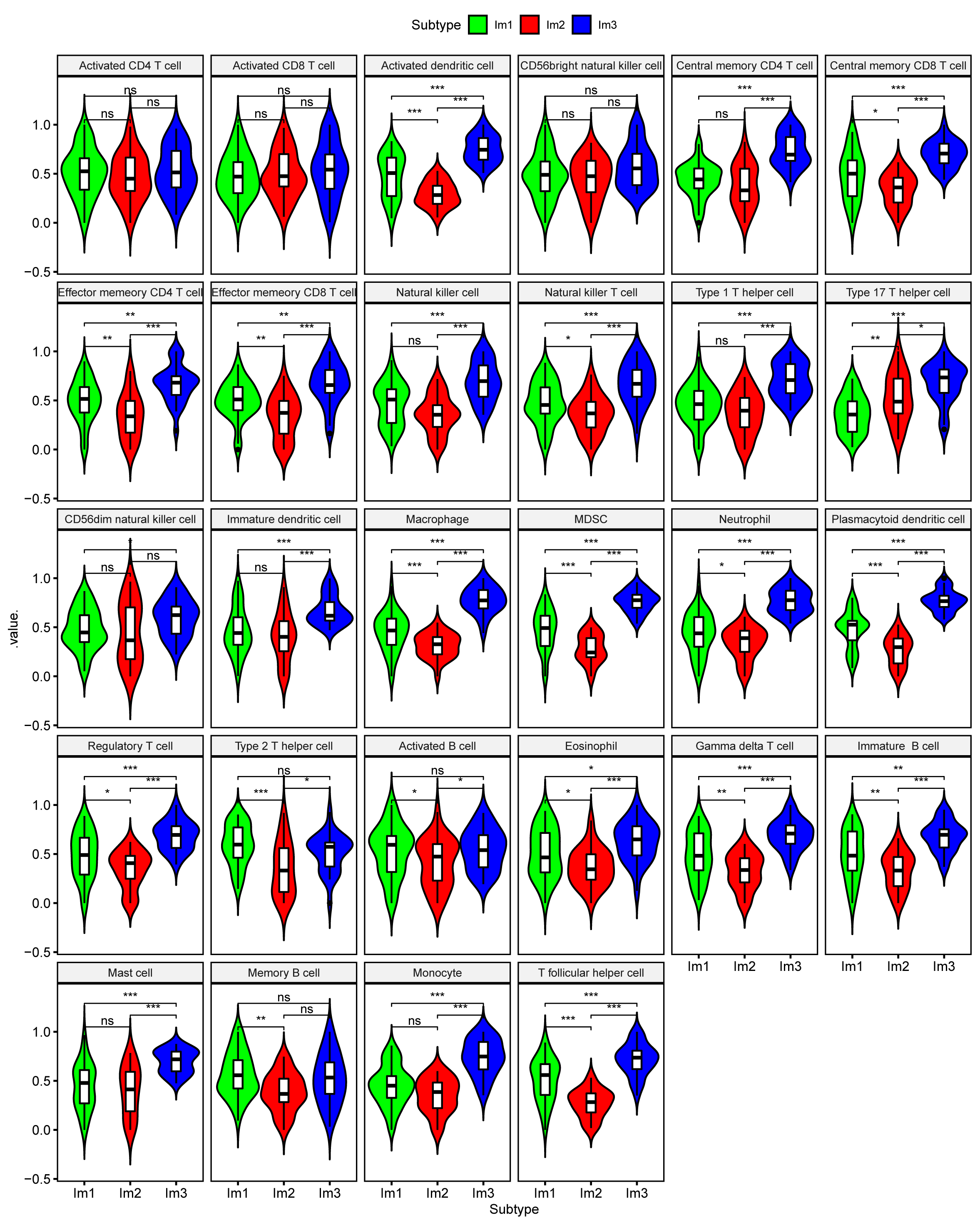


Figure S2. Infiltration of 28 immune cells in different clusters of TCGA-AML patients.


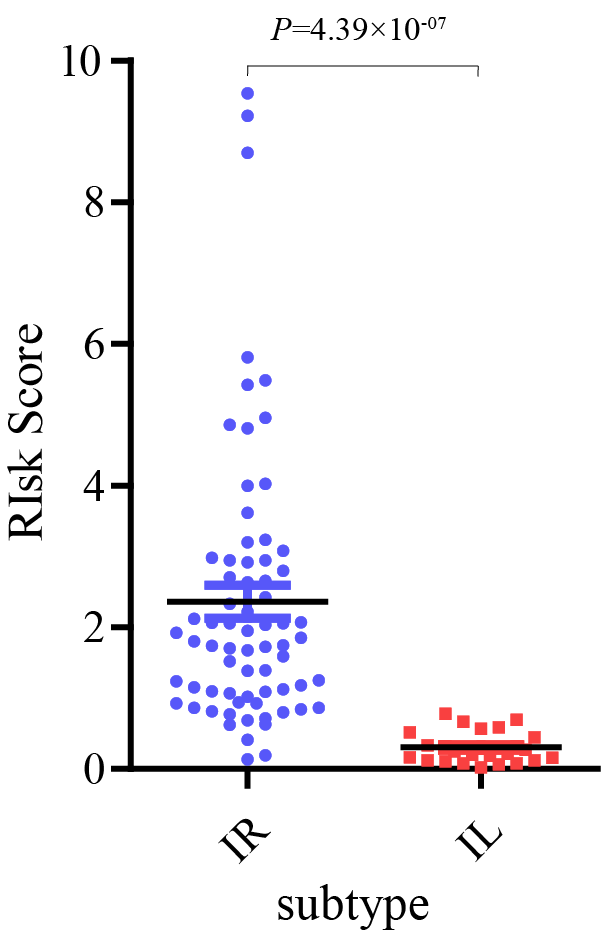


Figure S3. The risk score of IL and IR type.


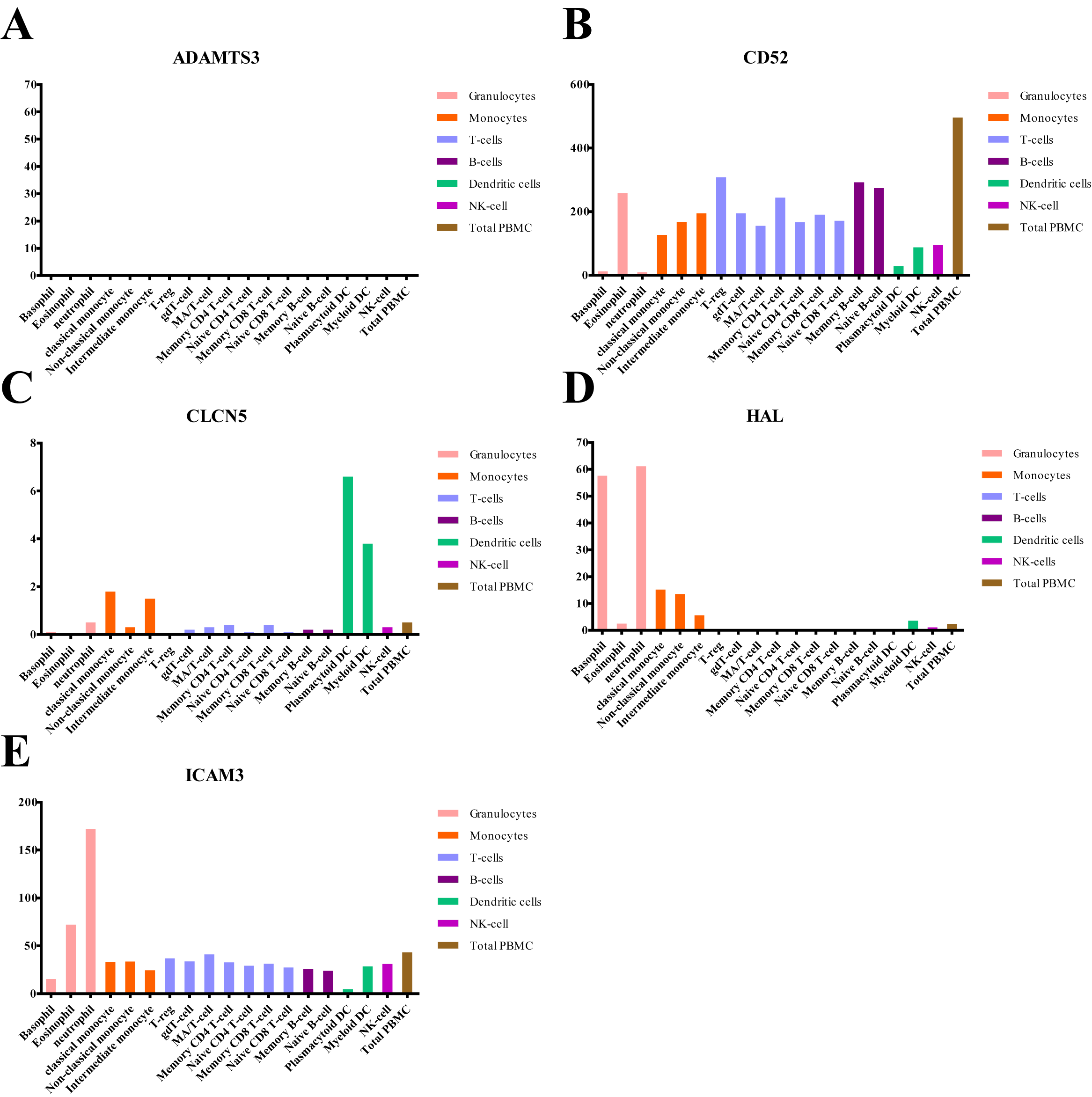


Figure S4. The expression value of 5 hub genes in various immune cells.

**Table S1.** clinical characters of TCGA-AML patients.

| ID | Gender | Age | OS | Status | FAB Category |
| --- | --- | --- | --- | --- | --- |
| TCGA-AB-2808 | male | 23 | 95.4 | alive | M2 |
| TCGA-AB-2819 | female | 52 | 83.2 | alive | M2 |
| TCGA-AB-2821 | male | 64 | 27.4 | dead | M1 |
| TCGA-AB-2828 | male | 55 | 76.1 | alive | M4 |
| TCGA-AB-2835 | male | 48 | 55.8 | alive | M5 |
| TCGA-AB-2846 | female | 57 | 46.7 | dead | M4 |
| TCGA-AB-2847 | male | 53 | 20.3 | dead | M1 |
| TCGA-AB-2849 | male | 39 | 74.0 | alive | M0 |
| TCGA-AB-2862 | female | 33 | 47.7 | alive | M3 |
| TCGA-AB-2863 | male | 63 | 1.0 | dead | M1 |
| TCGA-AB-2869 | female | 64 | 8.1 | alive | M2 |
| TCGA-AB-2870 | male | 76 | 5.1 | dead | M1 |
| TCGA-AB-2871 | male | 51 | 5.1 | alive | M1 |
| TCGA-AB-2872 | male | 42 | 21.3 | alive | M3 |
| TCGA-AB-2873 | female | 51 | 9.1 | alive | M5 |
| TCGA-AB-2874 | male | 59 | 13.2 | alive | M2 |
| TCGA-AB-2875 | male | 43 | 7.1 | alive | M2 |
| TCGA-AB-2876 | female | 45 | 40.6 | alive | M2 |
| TCGA-AB-2877 | female | 60 | 21.3 | alive | M1 |
| TCGA-AB-2878 | female | 47 | 12.2 | dead | M2 |
| TCGA-AB-2880 | male | 24 | 14.1 | dead | M1 |
| TCGA-AB-2881 | female | 48 | 13.1 | alive | M1 |
| TCGA-AB-2882 | female | 73 | 12.2 | dead | M2 |
| TCGA-AB-2883 | male | 60 | 24.3 | alive | M5 |
| TCGA-AB-2884 | female | 44 | 24.4 | dead | M1 |
| TCGA-AB-2885 | male | 71 | 7.1 | dead | M0 |
| TCGA-AB-2886 | male | 26 | 6.0 | alive | M2 |
| TCGA-AB-2888 | male | 57 | 11.1 | alive | M4 |
| TCGA-AB-2889 | male | 55 | 10.1 | alive | M4 |
| TCGA-AB-2892 | female | 42 | 31.4 | alive | M4 |
| TCGA-AB-2894 | female | 50 | 6.0 | dead | M5 |
| TCGA-AB-2895 | female | 41 | 5.1 | dead | M1 |
| TCGA-AB-2896 | female | 21 | 7.1 | dead | M5 |
| TCGA-AB-2897 | female | 50 | 8.1 | alive | M3 |
| TCGA-AB-2898 | female | 69 | 13.1 | alive | M1 |
| TCGA-AB-2899 | female | 76 | 22.4 | dead | M4 |
| TCGA-AB-2900 | male | 70 | 6.1 | dead | M1 |
| TCGA-AB-2901 | male | 27 | 2.0 | alive | M2 |
| TCGA-AB-2908 | male | 81 | 1.0 | dead | M2 |
| TCGA-AB-2911 | female | 51 | 39.5 | alive | M4 |
| TCGA-AB-2912 | male | 63 | 9.1 | dead | M4 |
| TCGA-AB-2913 | male | 61 | 40.5 | alive | M0 |
| TCGA-AB-2914 | female | 22 | 26.4 | alive | M2 |
| TCGA-AB-2916 | female | 48 | 29.4 | alive | M4 |
| TCGA-AB-2917 | female | 41 | 40.5 | alive | M0 |
| TCGA-AB-2919 | female | 54 | 2.0 | alive | M1 |
| TCGA-AB-2920 | male | 44 | 12.2 | dead | M2 |
| TCGA-AB-2924 | male | 59 | 3.0 | alive | M4 |
| TCGA-AB-2925 | male | 57 | 8.1 | dead | M5 |
| TCGA-AB-2927 | female | 88 | 3.0 | dead | M1 |
| TCGA-AB-2928 | female | 43 | 2.0 | dead | M1 |
| TCGA-AB-2929 | female | 71 | 4.1 | dead | M0 |
| TCGA-AB-2933 | male | 58 | 4.1 | dead | M4 |
| TCGA-AB-2934 | male | 65 | 0.9 | alive | M1 |
| TCGA-AB-2935 | male | 66 | 2.0 | dead | M4 |
| TCGA-AB-2936 | female | 61 | 2.0 | alive | M0 |
| TCGA-AB-2937 | female | 36 | 7.2 | dead | M1 |
| TCGA-AB-2939 | male | 72 | 15.2 | alive | M1 |
| TCGA-AB-2942 | female | 67 | 21.4 | alive | M4 |
| TCGA-AB-2948 | male | 67 | 19.3 | dead | M4 |
| TCGA-AB-2949 | male | 58 | 23.3 | alive | M0 |
| TCGA-AB-2950 | female | 34 | 10.2 | alive | M2 |
| TCGA-AB-2952 | female | 60 | 1.0 | dead | M1 |
| TCGA-AB-2955 | female | 56 | 16.3 | dead | M2 |
| TCGA-AB-2956 | male | 61 | 6.1 | dead | M5 |
| TCGA-AB-2959 | male | 71 | 16.3 | dead | M0 |
| TCGA-AB-2963 | male | 56 | 54.7 | dead | M1 |
| TCGA-AB-2965 | male | 60 | 11.2 | dead | M4 |
| TCGA-AB-2966 | female | 57 | 28.5 | dead | M2 |
| TCGA-AB-2970 | female | 34 | 10.2 | dead | M4 |
| TCGA-AB-2971 | female | 76 | 26.4 | dead | M4 |
| TCGA-AB-2973 | female | 68 | 20.3 | dead | M4 |
| TCGA-AB-2976 | male | 53 | 30.5 | dead | M1 |
| TCGA-AB-2977 | female | 71 | 1.0 | dead | M2 |
| TCGA-AB-2979 | female | 30 | 22.4 | alive | M1 |
| TCGA-AB-2980 | male | 50 | 23.3 | alive | M3 |
| TCGA-AB-2981 | female | 35 | 16.2 | alive | M5 |
| TCGA-AB-2982 | female | 29 | 5.0 | alive | M3 |
| TCGA-AB-2983 | male | 45 | 11.2 | dead | M0 |
| TCGA-AB-2984 | male | 38 | 38.6 | alive | M1 |
| TCGA-AB-2986 | female | 31 | 7.1 | dead | M2 |
| TCGA-AB-2987 | female | 75 | 6.1 | dead | M5 |
| TCGA-AB-2988 | female | 67 | 1.0 | dead | M1 |
| TCGA-AB-2990 | male | 51 | 15.2 | alive | M1 |
| TCGA-AB-2991 | female | 40 | 60.9 | alive | M3 |
| TCGA-AB-2992 | female | 32 | 56.9 | dead | M1 |
| TCGA-AB-2995 | male | 63 | 51.7 | alive | M2 |
| TCGA-AB-2996 | male | 74 | 52.7 | alive | M2 |
| TCGA-AB-2998 | female | 68 | 1.0 | dead | M3 |
| TCGA-AB-2999 | male | 62 | 57.8 | alive | M3 |
| TCGA-AB-3001 | female | 31 | 52.7 | alive | M3 |
| TCGA-AB-3002 | male | 68 | 47.7 | dead | M2 |
| TCGA-AB-3007 | male | 35 | 52.7 | alive | M3 |
| TCGA-AB-3008 | male | 22 | 27.4 | dead | M1 |
| TCGA-AB-3009 | male | 23 | 19.2 | dead | M4 |
| TCGA-AB-3011 | female | 21 | 62.8 | alive | M1 |
| TCGA-AB-3012 | male | 53 | 62.9 | alive | M3 |

**Table S2.** 98 survival-related immune genes (SIGs).

| 98 SIGs | p-value |
| --- | --- |
| ADAM12 | 0.009055077 |
| ADAMTS3 | 0.019672714 |
| ADGRE5 | 0.030478533 |
| AHNAK | 0.018578223 |
| AIF1 | 0.01115535 |
| AKT3 | 0.029314184 |
| ANXA5 | 0.041670163 |
| APOL3 | 0.012945343 |
| ARHGAP10 | 0.003128417 |
| ARL1 | 0.019752628 |
| ASB2 | 0.005555865 |
| ATP6V1A | 0.040023492 |
| BST2 | 0.017630636 |
| C1orf162 | 0.024263467 |
| C1orf54 | 0.014255787 |
| CCR5 | 0.013876728 |
| CD109 | 0.012251905 |
| CD37 | 0.000151561 |
| CD4 | 0.013047345 |
| CD52 | 0.000148262 |
| CD79B | 0.047103955 |
| CFL1 | 0.028244461 |
| CLCN5 | 0.0000025 |
| CLIC2 | 0.012485077 |
| CLIC3 | 0.025449448 |
| CREB5 | 0.024746533 |
| CRYBB1 | 0.031592817 |
| CSF2RA | 0.040604671 |
| CTPS1 | 0.006880889 |
| CTSD | 0.016154468 |
| CXCR2 | 0.039440985 |
| CYP27A1 | 0.032368574 |
| CYTH1 | 0.010866987 |
| DAB2 | 0.030817755 |
| DAPK1 | 0.046970159 |
| DARS | 0.009274375 |
| DAXX | 0.000084 |
| DPYD | 0.013317327 |
| EMP3 | 0.007094884 |
| F12 | 0.03259722 |
| FABP5 | 0.004115137 |
| FAM49A | 0.021102459 |
| FERMT3 | 0.000482707 |
| GAB3 | 0.012790197 |
| HAL | 0.030493772 |
| HCP5 | 0.005447746 |
| HELLS | 0.030883597 |
| HNMT | 0.016554784 |
| HRH1 | 0.015225832 |
| HSPA6 | 0.030522513 |
| ICAM3 | 0.048885585 |
| IDH3A | 0.034302608 |
| IGFBP5 | 0.031599509 |
| IKZF1 | 0.03136533 |
| IL3RA | 0.030153684 |
| INPP5F | 0.003711848 |
| ITGAL | 0.002788804 |
| ITGAM | 0.036646269 |
| ITGB2 | 0.030734879 |
| ITGB4 | 0.020982102 |
| KIR2DL1 | 0.011560605 |
| KIR3DL2 | 0.048387167 |
| KLRG1 | 0.015460397 |
| LAMP2 | 0.013655645 |
| LGALS1 | 0.000181036 |
| LRRC42 | 0.020273562 |
| LSP1 | 0.000744407 |
| LST1 | 0.015009468 |
| MAK | 0.030356179 |
| MBP | 0.038905732 |
| MMD | 0.017479911 |
| MOCOS | 0.004815371 |
| NCAM1 | 0.009065868 |
| NUCB2 | 0.00788809 |
| OFD1 | 0.022159756 |
| PDCD1 | 0.021997507 |
| PIK3IP1 | 0.006985001 |
| PLA2G4A | 0.033592713 |
| PNPLA6 | 0.007064373 |
| PTGES2 | 0.035224203 |
| PTGS1 | 0.002302456 |
| RPS9 | 0.026383806 |
| RUBCN | 0.001673805 |
| S100A4 | 0.000139415 |
| SELE | 0.004005489 |
| SFXN3 | 0.001168979 |
| SIGLEC10 | 0.036557072 |
| SLA | 0.027371484 |
| SPCS3 | 0.033123296 |
| ST3GAL6 | 0.022176552 |
| ST8SIA4 | 0.045818496 |
| TACSTD2 | 0.025528048 |
| TLR9 | 0.03758144 |
| TNFAIP2 | 0.001039854 |
| TOGARAM2 | 0.030739985 |
| TOX4 | 0.034453285 |
| UPP1 | 0.046621377 |
| VCAM1 | 0.030889137 |
